# Effects of Inhaled Amitriptyline on Airway Function and Immune Responses in Experimental Asthma

**DOI:** 10.3390/arm94040058

**Published:** 2026-08-06

**Authors:** Anna Michely, Svenja Böll, Lida Yao, Regina Ben Hamza, Irina Rachimow, Klaus Tenbrock, Christian Martin, Eva Verjans

**Affiliations:** 1 Department of Pediatrics, Medical Faculty, RWTH Aachen, University Hospital Aachen, 52074 Aachen, Germany; 2 Institute of Pharmacology and Toxicology, Medical Faculty, RWTH Aachen, University Hospital Aachen, 52074 Aachen, Germany

**Keywords:** bronchial asthma, amitriptyline, airway hyperresponsiveness, type 2 inflammation, bronchodilation

## Abstract

**Highlights:**

**What are the main findings?**

- Inhaled amitriptyline improves lung function in experimental allergic airway inflammation (AAI) by reducing airway obstruction.
- Amitriptyline exerts context-dependent immunomodulatory effects that vary between experimental models, with anti-inflammatory effects observed in vivo in the OVA-induced AAI model but not in the HDM model or in differentiated human T_H_2 cells in vitro.

**What are the implications of the main findings?**

- Inhaled amitriptyline represents a promising therapeutic candidate for improving lung function in AAI.
- Further studies are needed to elucidate the cellular and molecular mechanisms underlying the context-dependent immunomodulatory effects of amitriptyline.

**Abstract:**

Background: Bronchial asthma is a chronic inflammatory airway disease characterized by acute bronchoconstriction and type 2-driven inflammation. This study investigated whether inhaled amitriptyline, a functional inhibitor of acid sphingomyelinase, exerts both bronchodilatory and immunomodulatory effects in experimental murine models of allergic airway inflammation (AAI) and human cellular systems. Methods: Acute AAI was induced in mice using ovalbumin (OVA) and house dust mite (HDM) protocols, respectively. Inhaled amitriptyline (3.3 mg/mL) was administered for either 20 days (short-term) or 36 days (long-term). Lung function was assessed using FlexiVent^®^, and inflammatory markers including IgE, eosinophils, and type 2 cytokines were measured in bronchoalveolar lavage fluid and lung tissue. Complementary experiments were included using passively sensitized PCLSs and human type 2-differentiated CD4^+^ T cells. Results: Inhaled amitriptyline improved lung mechanics in both the OVA and HDM models, reducing total respiratory resistance and elastance. In the OVA model, eosinophil and T cell counts in BALF were decreased, whereas immunomodulatory effects were less pronounced in the short-term HDM model. In human T_H_2 cells, no significant changes in cytokine production or gene expression were observed. Ex vivo, amitriptyline dose-dependently inhibited allergen-induced bronchoconstriction in PCLSs. Conclusions: Inhaled amitriptyline improves lung function across murine models of AAI, supporting its potential in exhibiting model-dependent immunomodulatory effects, and directly attenuates allergen-induced bronchoconstriction, supporting its potential as a bronchodilator with context-dependent immunomodulatory properties.

## 1. Introduction

Bronchial asthma is a chronic inflammatory disease with an increasing prevalence worldwide, affecting up to 300 million people [1,2]. It typically manifests in childhood and is characterized by airway inflammation, edema formation, and increased mucus production, leading to acute bronchial hyperreactivity, resulting in tachydyspnea, cough, and restlessness [3]. Immunologically, bronchial asthma is predominantly a type 2-driven inflammatory disease, associated with increased migration of eosinophilic granulocytes to the site of inflammation, increased IgE serum levels, and enhanced production of the key cytokines IL-4, IL-5, and IL-13 [4]. IL-4 is a central cytokine in the development of allergic asthma. It promotes differentiation of naïve CD4^+^ T cells into T_H_2 cells, induces immunoglobulin E (IgE) class switching in B cells, and amplifies type 2 immune responses, thereby contributing to eosinophilic airway inflammation and airway hyperresponsiveness [5]. In addition to T_H_2 lymphocytes, innate immune cells such as basophils and group 2 innate lymphoid cells (ILC2s) are important sources of IL-4 and contribute to the initiation and maintenance of allergic airway inflammation. These cell populations play important roles in shaping adaptive type 2 immune responses and may represent additional targets for immunomodulatory therapies [6,7,8]. Current standard therapies combine short-acting β2-adrenergic agonists and inhaled corticosteroids for baseline disease control with long-term treatment, including biologicals such as IL-4R antibodies, anti-IL-5, and anti-IgE for severe cases. While biologicals target key components of the type 2-driven immune response, they do not address acute bronchoconstriction. Short-acting β2-adrenergic agonists provide only short-term symptomatic bronchodilation without modulating the underlying immunopathology [9]. This highlights the need for novel medications that effectively modulate both aspects. Amitriptyline may offer such a dual therapeutic approach, with a long-lasting bronchodilatory effect and fewer side effects than existing short-acting β2-adrenergic agonists.

Amitriptyline, a tricyclic antidepressant, was already investigated in the 1970s for potential benefits in bronchial asthma [10,11]. Early studies suggested that amitriptyline might influence asthma pathophysiology. Over time, however, amitriptyline as a potential medication lost relevance, with most studies focusing on direct receptor binding rather than direct effects on bronchoconstriction or inflammation [12,13]. Scientific interest in amitriptyline was recently renewed when it was identified as a functional inhibitor of acid sphingomyelinase (ASM), a key enzyme involved in sphingolipid metabolism [14].

More recently, research has revealed that the knockout of acid sphingomyelinase (ASM) has an immunomodulatory effect [15,16,17,18]. ASM knockout reduces typical type 2 cytokines in T lymphocytes of asthmatic mice, decreases bronchial hyperresponsiveness, and reduces the numbers of eosinophilic granulocytes and T_H_2 lymphocytes in bronchoalveolar lavage fluid and lung tissue, highlighting the importance of this pathway in allergic airway inflammation [16]. Further, amitriptyline has been reported to function as an inhibitor of bronchoconstriction in different ex vivo models and to induce effective relaxation of pre-constricted airways, with evidence suggesting that this bronchodilatory action may occur independent of ASM inhibition [19]. Preliminary clinical observations in small patient cohorts further suggest the potential benefit of amitriptyline in asthma. In these cohorts, systemic administration of amitriptyline was associated with clinical improvement [20]. In patients with cystic fibrosis, inhaled amitriptyline led to reduced IL-1β levels compared to untreated controls and resulted in improvement of lung function parameters like FEV1 [21].

These findings led to the hypothesis that inhaled amitriptyline might be the first therapeutic agent that has potent effects on both the type 2-allergic phenotype and on acute bronchoconstriction in asthma.

In a 35-day ovalbumin murine model of AAI, we investigated the effect of amitriptyline on airway hyperresponsiveness (AHR) as well as on key cytokines and immune cells relevant to bronchial asthma. While food-allergen-linked OVA-induced murine models of AAI are commonly used for experimental asthma, we additionally employed a 26-day house dust mite (HDM) murine model of AAI as an inhaled allergen to better reflect clinically relevant allergic responses [22]. The present study focused on experimental models of predominantly type 2 AAI. The OVA model induces a classical eosinophilic type 2 immune response, whereas the HDM model more closely resembles clinically relevant AAI and may additionally involve more heterogenous inflammatory pathways while remaining largely type 2-driven [23,24,25,26].

Historical case reports have suggested that amitriptyline may influence asthma symptoms in humans [11]. To follow up on these observations, we additionally examined the effects of amitriptyline on differentiated T_H_2 cells isolated from human blood. Furthermore, the impact of amitriptyline on airway responses during the early-phase response of asthma was investigated using ex vivo rat precision-cut lung slices that were passively sensitized with ovalbumin.

Focusing on both immunological and non-immunological mechanisms, this study explored the potential therapeutic application of inhaled amitriptyline in bronchial asthma.

## 2. Materials and Methods

### 2.1. Animal and Human T-Lymphocytes

Experiments were performed with 6- to 12-week-old C57BL/6 wild-type mice and 6- to 10-week-old rats obtained from Janvier Labs (Le Genest-Saint-Isle, France). The regional governmental authorities approved the study, and animal procedures were performed in accordance with the German animal protection law (AZ 81-02-04-2018.A095 (in vivo OVA model and in vivo HDM model, approval date: 14 January 2019); AZ 2025-32 (in vivo HDM model, approval date: 28 July 2025); A4-30332A4 (ex vivo PCLS, approval date: 22 May 2023); AZ 87-51.04.2010.A247 (passive rat sensitization, approval date: 5 November 2010)).

Human cellular components were prepared from human whole-blood healthy volunteers. The study was approved by the local ethics committee of the Medical Faculty Aachen, RWTH Aachen (EK 24-406, approval date: 25 January 2025). All volunteers gave written informed consent.

### 2.2. Experimental Design of Ovalbumin (OVA) Sensitization

**Figure A1:**
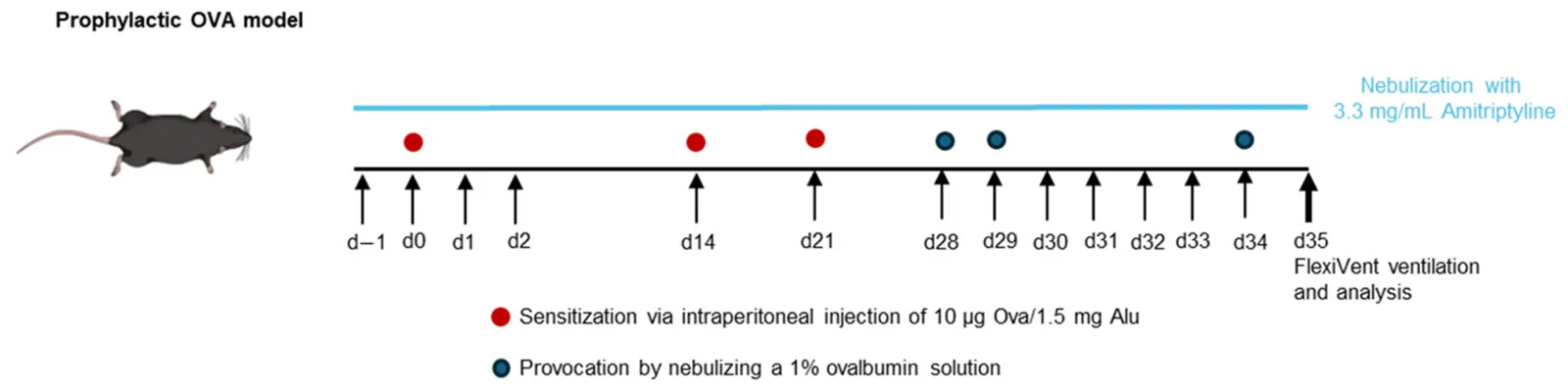
Experimental design of ovalbumin (OVA) sensitization. Mouse icon adapted from Mind the Graph (licensed under CC BY-SA 4.0, https://creativecommons.org/licenses/by-sa/4.0/, accessed 6 August 2026). All remaining figure elements were created by the authors.

Mice were injected i.p. with OVA (10 µg) and aluminum hydroxide (Alu) (1.5 mg) on days 0, 14, and 21 in a total volume of 200 µL. Sensitized mice were then exposed to nebulized OVA (1%) via a PARI TurboBOY^®^ SX (PARI GmbH, Starnberg, Germany) on days 28, 29, and 34 for 30 min. Starting one day prior to the first sensitization until day 35, mice were additionally nebulized with 3.3 mg/mL amitriptyline for 20 min. Control animals were treated with 1.5 mg Alu i.p. followed by inhalation with 0.9% NaCl. On day 35, mice were mechanically ventilated with the FlexiVent^®^ ventilator (SIREQ Scientific Respiratory Equipment Inc., Montreal, QC, Canada), and analyses of lung, spleen, serum, and bronchoalveolar lavage fluid (BALF) were performed (see Appendix A, Figure A1).

### 2.3. Experimental Design of House Dust Mite Sensitization

**Figure A2:**
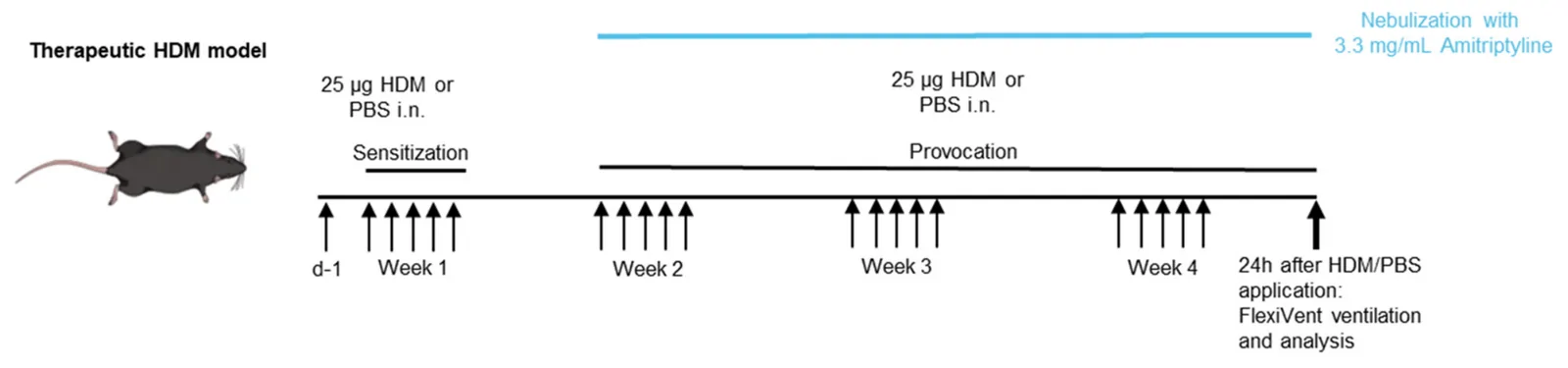
Experimental design of house dust mite (HDM) sensitization. Mouse icon adapted from Mind the Graph (licensed under CC BY-SA 4.0, https://creativecommons.org/licenses/by-sa/4.0/, accessed 6 August 2026). All remaining figure elements were created by the authors.

Mice were treated with 25 µg of house dust mite extract (Dust Mite Extract from *Dermatophagoides pteronyssinus* (Chondrex Inc., Woodinville, WA, USA) in a total volume of 40 µL phosphate-buffered saline (PBS) 5 times a week intranasally for 4 weeks. Mice were anesthetized and treated with 5% isoflurane by inhalation. Starting on day 7, mice were exposed to nebulized amitriptyline (3.3 mg/mL) via a PARI TurboBOY^®^ SX for 30 min until day 26. Control animals were treated with PBS intranasally and were nebulized with NaCl (0.9%). On day 26, mice were mechanically ventilated with a FlexiVent^®^ ventilator (SIREQ Scientific Respiratory Equipment Inc., Montreal, Canada), and analyses of lung, spleen, serum, and bronchoalveolar lavage fluid (BALF) were performed (see Appendix A, Figure A2).

### 2.4. Cytokine Measurements and RT-qPCR Analysis

For the analysis of the ovalbumin and house dust mite sensitization, murine IgE, IL-5, and IL-4 were quantified using enzyme-linked immunosorbent assay (ELISA) kits (R&D Systems Inc., Minneapolis, USA) in lysed lung cells and BALF according to the manufacturer’s instructions. IL-2, IL-4, and IL-5 were measured in supernatants of differentiated human T cells (R&D Systems). Lung tissue was pulverized, homogenized in lysis buffer and centrifuged at 13,000× *g* for 10 min at 4 °C. Supernatants were used for ELISA measurements. Cytokine concentrations were normalized to the initial weight of the lung tissue used.

Gene expression of *Il5*, *Il2*, *Il4*, *Il4R*, and *Il13* (Table A1) of differentiated human T cells was quantified by reverse transcription (RT)-qPCR analysis using the CFX connect detection system (Bio-Rad, Hercules, CA, USA). All target genes were analyzed in technical triplicates, and data analysis was performed using CFX Maestro software (Version: 1.1, Bio-Rad, Hercules, CA, USA). Primer amplification efficiencies were assessed with LinReg PCR software (Version: 2020.2, SoftGuide GmbH & Co. KG, Wolfsburg, Germany), with an accepted range of 85–100%. Primers were designed across exon–exon boundaries to ensure specificity. No-RT and NTC controls were included for all genes. The reference genes *TATA-binding protein* (*TBP*) and *ribosomal protein L13* (*Rpl13*) were used to normalize the mRNA levels.

### 2.5. FlexiVent^®^ Ventilation

All mice were nebulized with 0.9% NaCl or 3.3 mg/mL amitriptyline in a chamber connected to a pariboy (red insert, size of nebulization droplets: 2.8 µm) directly before FlexiVent^®^ ventilation. Mice were anaesthetized with pentobarbital sodium (80 mg/kg) and fentanyl (0.04 mg/kg). Lidocaine (1%) was used as a local anesthetic. All mice were mechanically ventilated using the FlexiVent^®^ (SIREQ Scientific Respiratory Equipment Inc., Montreal, Canada) ventilation setup, with a tidal volume of 10 mL/kg and a positive end-expiratory pressure (PEEP) of 2cmH_2_O. Dynamic lung mechanics were measured by applying oscillatory waveforms and analyzed via the forced oscillation technique (FOT). Single-frequency forced oscillation (SnapShot-150) and broadband low-frequency forced oscillation (Quick Prime-3) maneuvers were sequentially executed. Total respiratory system resistance was calculated using FlexiVent^®^ software (FlexiWare^®^ 8.0.3, SIREQ, Canada). By fitting measured SnapShot data to a linear single-compartment model via multiple linear regression. Respiratory system input impedance was derived from the Quick Prime data, and tissue resistance was determined by iterative fitting of the constant-phase model to the impedance spectra.

After an initial period of basal ventilation lasting 30 min, airway hyperresponsiveness was induced by nebulized acetylcholine (ACh; 0–3.16 mg/mL). For each ACh concentration, lung function measurements were performed 12 times over a 3 min interval.

During the course of these physiological measurements, sample attrition occurred due to animal death in the OVA–amitriptyline groups as well as in the HDM–amitriptyline and HDM-NaCl group.

### 2.6. T Cell Isolation and T Cell Differentiation

Human T cells were isolated from human whole blood. Human T cells were isolated from whole blood using a sensity gradient matrix (Pancoll), and the lymphocyte fraction was isolated and further separated using a CD4+ CD25+ Regulatory T Cell Isolation Kit (Miltenyi). CD4+ CD25-cells were incubated with anti-CD3 and anti-CD28 antibodies and a differentiation supplement (STEMCELL Technologies, Vancouver, Canada). On day 6, cells were treated with different concentrations of amitriptyline (1 µM, 5 µM). After 7 days of differentiation, cell culture supernatant and lysed cells were used for ELISA, qPCR, and flow cytometer analyses.

**Figure A3:**
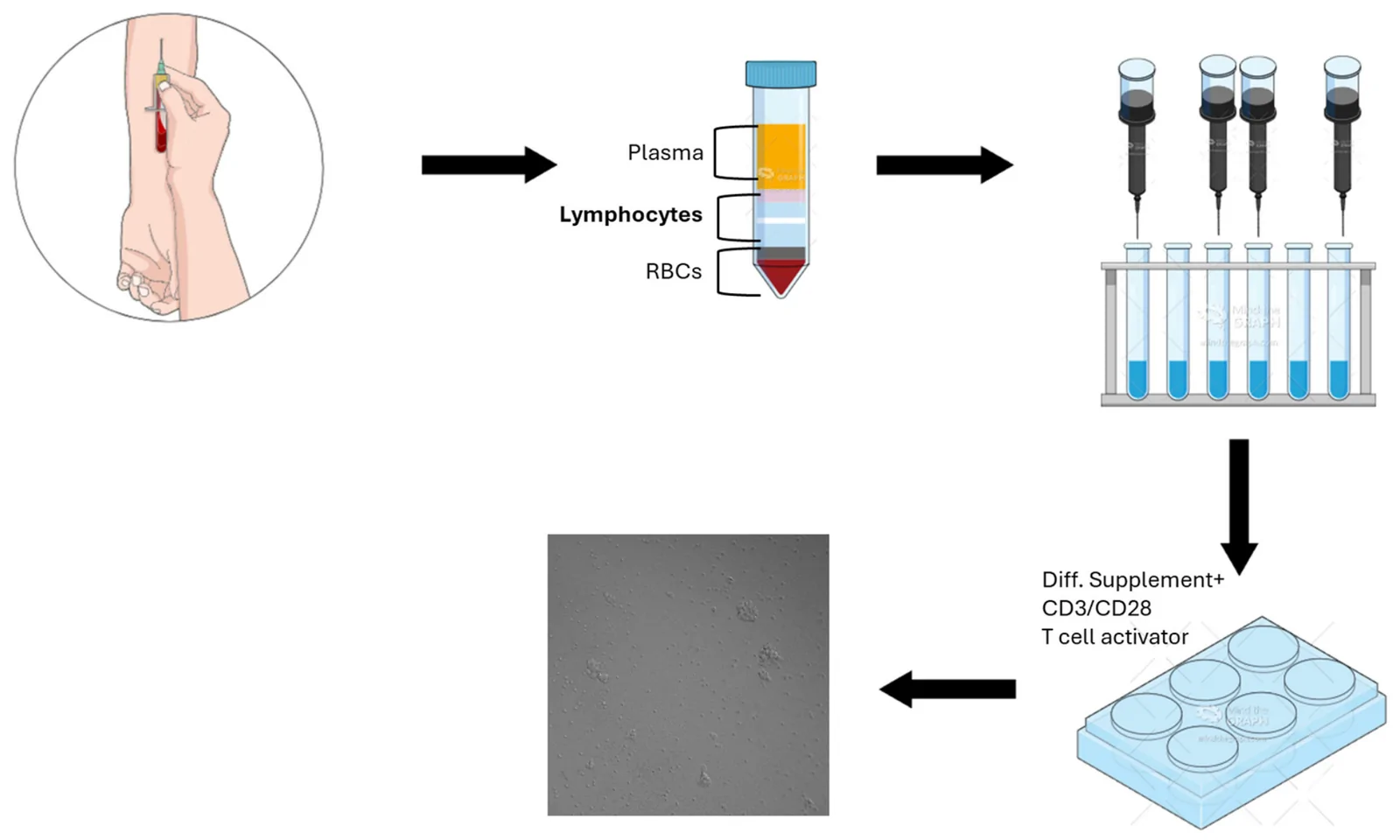
T cell isolation and T cell differentiation. Figure created using Mind the Graph (licensed under CC BY-SA 4.0, https://creativecommons.org/licenses/by-sa/4.0/, accessed 6 August 2026). Microscopy images were provided by the authors.

Cell viability was tested with an FITC Annexin V Apoptosis Detection Kit with 7-AAD (BioLegend, San Diego, CA, USA) (see Appendix A, Figure A3).

### 2.7. Precision-Cut Lung Slices

Precision-cut lung slices were prepared as described in [27,28]. Intraperitoneal anesthesia was performed with pentobarbital. Following the complete cessation of cardiac activity, the trachea of rats was cannulated, and the abdomen and diaphragm were opened up. The lungs were filled with 1.5% low melting agarose. Tissue cores were cut into slices between 250 and 300 µm with a Krumdieck tissue slicer (Alabama, Munford, AL, USA). To remove residual agarose, PCLSs were incubated at 37 °C with several media changes. At 24–48 h after the preparation, slices completely free from agarose with intact cilia beating and an intact smooth muscle layer were used for the following experiments.

### 2.8. Passive Sensitization

Precision-cut lung slices (PCLS) from rats were incubated overnight in medium containing 1% serum from either OVA-sensitized or non-sensitized rats [29] (animals were completely bled, and samples were stored at −80 °C for long-term storage). On the following day, the slices were pretreated for 30 min with different mediators. A combination of IBMX (1 mM, dissolved in DMSO) and salbutamol (1 mM, dissolved in EtOH) was used as a positive control for maximal dilatation. Amitriptyline was tested in two concentrations (1 µM and 5 µM). EtOH and DMSO were used as negative controls.

**Figure A4:**
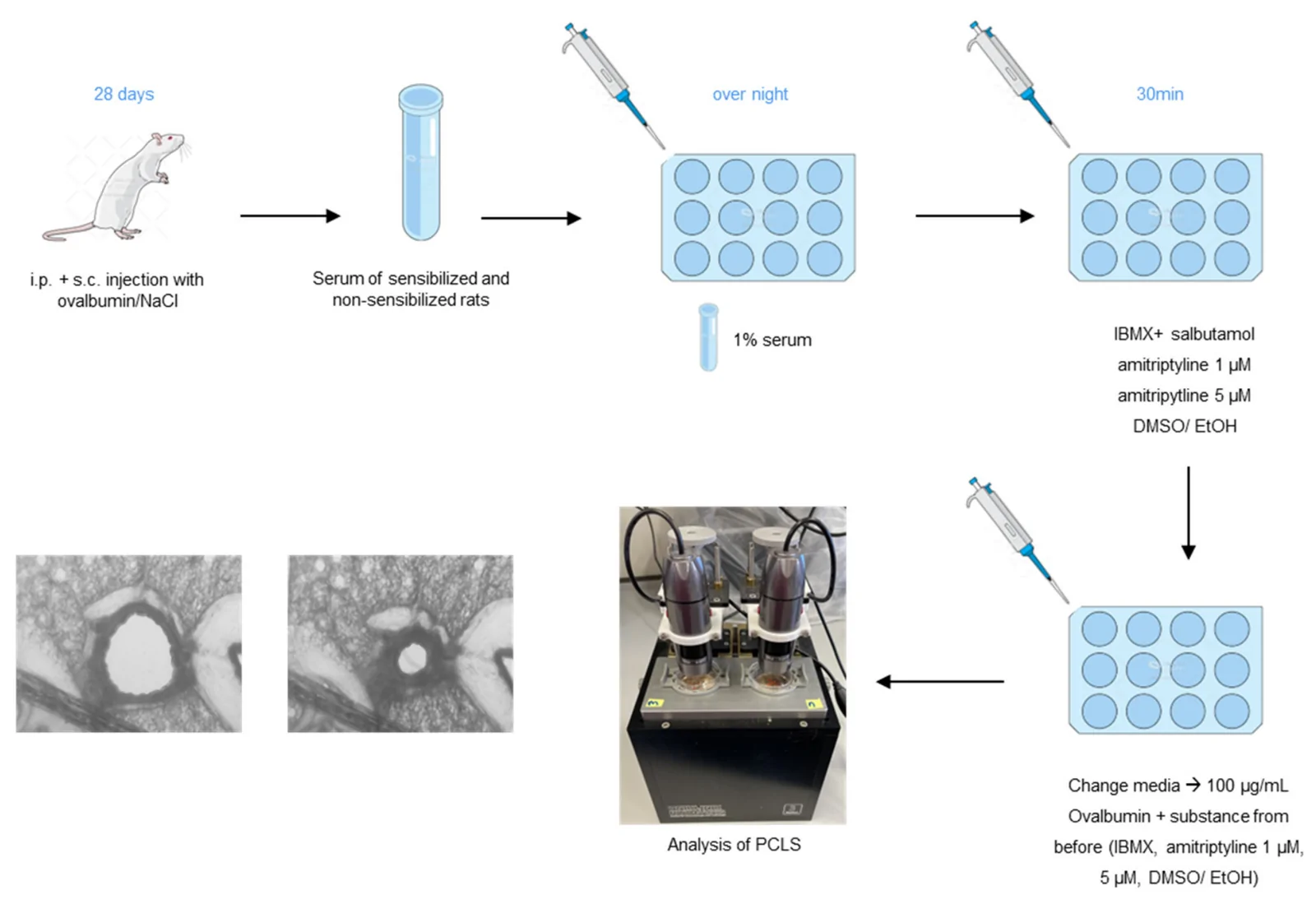
Experimental design of passive sensitization. Rat icon, sample tube icon, pipette icon and cell culture plate icon adapted from Mind the Graph (licensed under CC BY-SA 4.0, https://creativecommons.org/licenses/by-sa/4.0/, accessed 6 August 2026). All remaining figure elements were created by the authors.

After 30 min pretreatment, the medium was completely replaced, and slices were stimulated with 100 µg/mL ovalbumin as a trigger, together with the previously applied substances. For measurements, PCLS were placed into a culture dish, and slices were secured to the bottom using a custom platinum wire. Images were recorded every 10 s for 60 min with USB microscopes. The images were analyzed with ImageJ software (ImageJ 1.43 u, National Institutes of Health, Bethesda, MD, USA), taking the initial airway area as 100% (see Appendix A, Figure A4).

### 2.9. Statistical Analysis

Statistical analyses were performed using GraphPad Prism 9 (Graphpad Software, San Diego, CA, USA). Quantitative data are presented as mean ± standard error of the mean (SEM). Normality of data was assessed using the Shapiro–Wilk test. For parametric data, a two-tailed unpaired Student’s *t*-test was utilized. In addition, based on a directional hypothesis *a priori*, focusing exclusively on the effect of amitriptyline, the statistics for a one-tailed analysis are given in brackets {}. This decision was based on our previous studies demonstrating the protective effects of ASM inhibition in allergic asthma and consistent bronchodilatory effects of amitriptyline in experimental models [16,19]. Non-parametric data were analyzed using the one-tailed Mann–Whitney test. For comparison involving multiple groups, one-way analysis of variance (ANOVA) followed by Tukey’s multiple comparison test was used for parametric data, whereas the Kruskal–Wallis test followed by Dunn’s multiple comparison test was applied for nonparametric data. Differences in serum levels between OVA and HDM were analyzed using ANCOVA with experimental groups assigned as the fixed factor and animal body weight as the covariate; *p*-values < 0.05 were considered to be statistically significant (* *p* < 0.05, ** *p* < 0.01, and *** *p* < 0.001).

Sample size determination: Sample sizes for the in vivo OVA and HDM experiments were determined based on *a priori* power calculations (JMP 7.0.1 SAS Institute Inc., Cary, NC, USA, α = 0.05, power = 0.8). For the human T_H_2 cell and PCLS experiments, sample sizes were based on previous experience with these established experimental models and comparable studies from our laboratory. These experiments were designed as exploratory mechanistic studies, and no formal power calculation was performed.

## 3. Results

### 3.1. Inhalation of Amitriptyline Attenuates Airway Hyperresponsiveness in a Prophylactic OVA-Induced Murine Model of AAI

**Figure 1:**
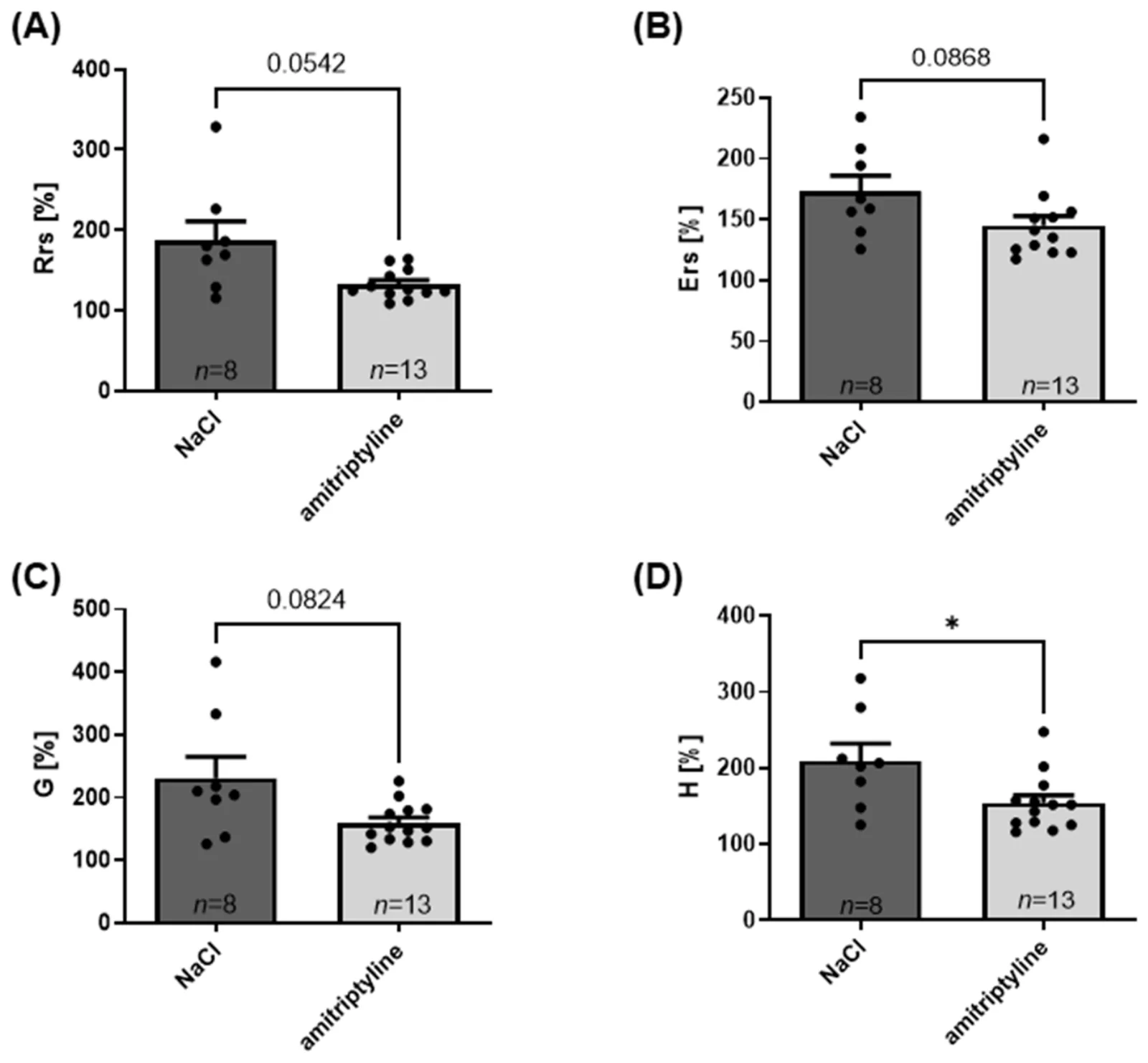
Lung function parameters of ovalbumin (OVA)-sensitized animals were measured with the FlexiVent^®^ system after application of 3.16 mg/mL acetylcholine (Ach). All physiological values were normalized on basal ventilation metrics. (**A**) Total lung resistance Rrs (*p* = 0.0542) {*p* = 0.007}, (**B**) elastance Ers (*p* = 0.0868) {*p* = 0.0320}, (**C**) tissue resistance G (*p* = 0.0824) {*p* = 0.0121}, and (**D**) tissue elastance H (*p* = 0.0497) {*p* = 0.0103} of OVA-sensitized mice, nebulized with 0.9% NaCl or 3.3 mg/mL amitriptyline, were measured. The data were tested for normal distribution and analyzed using a two-sided *t*-test. All graphs represent means ± SEM; * *p* ≤ 0.05. Each dot represents one mouse.

In previous studies, we demonstrated that acid sphingomyelinase knockout (ASM-KO) mice exhibit a protective phenotype in bronchial asthma [16]. Building on this, ex vivo experiments revealed that amitriptyline effectively inhibits bronchoconstriction but that these effects were ASM-independent [19]. Therefore, we investigated the effect of amitriptyline on lung function parameters in vivo in a prophylactic ovalbumin-induced murine model of AAI. Amitriptyline was administered daily for 35 days throughout both the sensitization and provocation phases (starting at day 1). WT mice treated with OVA and nebulized with amitriptyline and controls were ventilated using the FlexiVent^®^ setup to assess airway hyperresponsiveness (AHR). Total respiratory resistance (Rrs), total elastance (Ers) of the airways, and tissue resistance (G), which characterizes the smaller airways, decreased in the amitriptyline group compared to controls (Figure 1). Using a two-sided statistical test, only the reduction in tissue elastance (H) reached statistical significance. However, based on our *a priori* hypothesis of a protective effect of amitriptyline on airway function, a one-sided *t*-test was performed, showing significant reductions in all four parameters. These findings demonstrate that inhalation of amitriptyline during the sensitization and provocation phase protects against AHR during OVA-induced asthma exacerbation.

### 3.2. Inhaled Amitriptyline Does Not Induce Systemic Weight Gain and Modulates Eosinophilic Infiltration in the OVA Model

**Figure 2:**
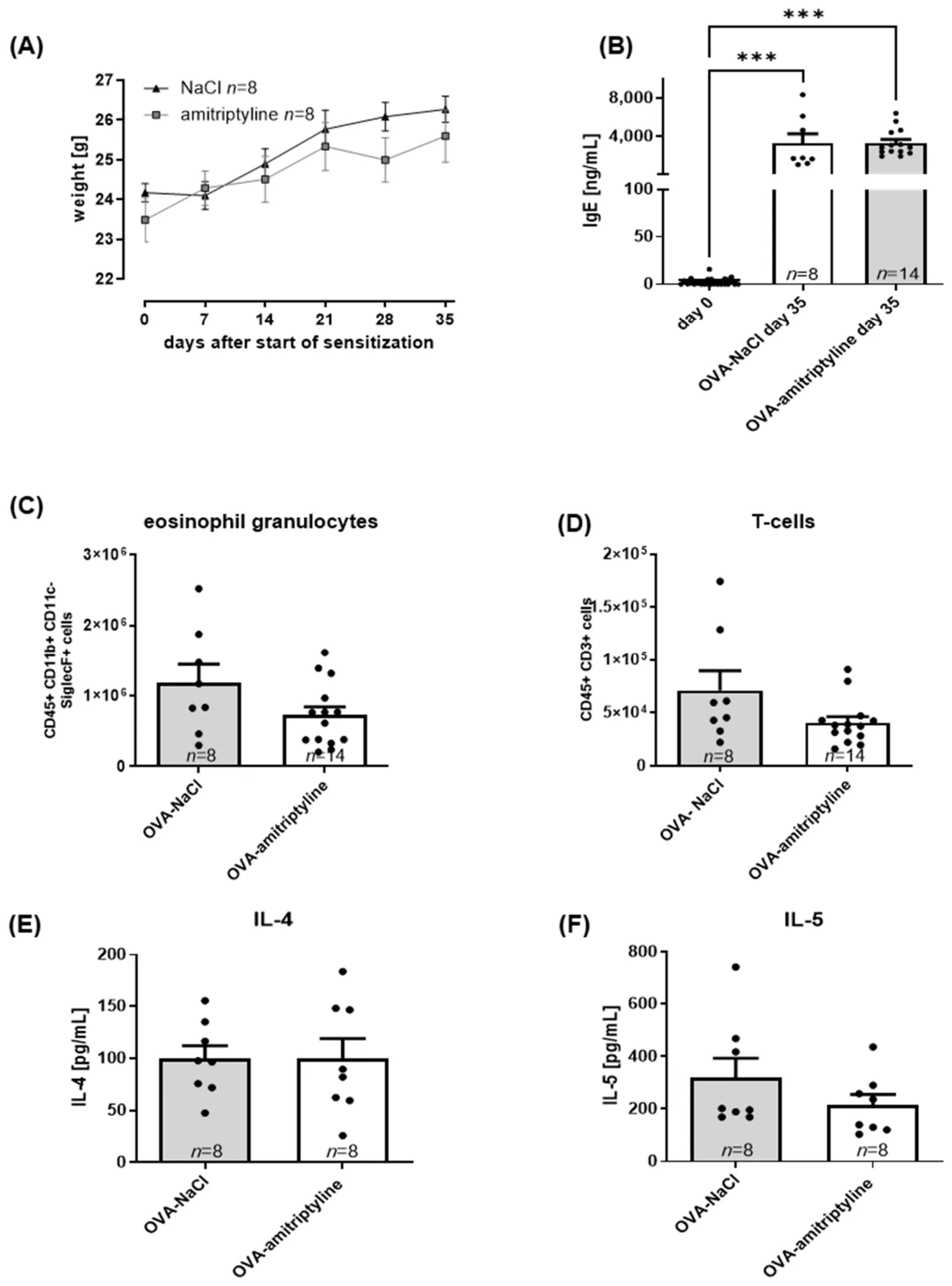
Immunomodulatory effects of amitriptyline in the ovalbumin (OVA)-induced murine model of AAI. (**A**) Effect of inhaled amitriptyline on the weight of animals during the time of sensitization (*p* = 0.8692). (**B**) Significant increase in IgE serum levels of OVA-sensitized mice (*p* = <0.001). (**C**) Counts of eosinophilic granulocytes (*p* = 0.1439) {*p* = 0.0426} and (**D**) T cells in the bronchoalveolar lavage fluid (BALF) of OVA-sensitized animals ± amitriptyline (*p* = 0.1579) {*p* = 0.0475}. (**E**) IL-4 (*p* = 0.9930) {*p* = 0.4965} and (**F**) IL-5 (*p* = 0.2376) {*p* = 0.1159} cytokine levels of the BALF. One animal in the OVA–amitriptyline group died during the FlexiVent^®^ lung function measurements. Therefore, lung function data include n = 13, whereas lung tissue and serum analyses include n = 14, as tissue and blood samples from this animal were collected and included in the respective analyses. The data were tested for normality: (**A**) was analyzed using a two-way ANOVA, (**B**) was analyzed using a one-way ANOVA, and (**C**–**F**) were analyzed using a two-sided *t*-test. All graphs represent means ± SEM, *** *p* ≤ 0.001. Each dot represents one mouse.

In clinical practice, and in contrast to our study, amitriptyline is often administered orally, and this is associated with the side effect of weight gain [15,30]. Inhaled amitriptyline led to no changes in body weight in amitriptyline-treated mice versus controls (Figure 2A). Body weight did not differ between the two groups. Thus, inhalation of amitriptyline up to 35 days did not induce characteristic weight gain, which is likely associated with reduced systemic absorption in the bloodstream compared to oral treatment.

To further characterize inflammatory responses in the ovalbumin-induced murine model of AAI, IgE-ELISA and flow cytometry of BALF were performed. Successful induction by AAI was confirmed by elevated serum IgE levels. OVA-sensitized animals showed significantly increased IgE serum levels at day 35 compared to the beginning of sensitization, but levels of amitriptyline-treated animals and controls were the same (Figure 2B). In contrast, eosinophilic granulocytes (Figure 2C) and T cells (Figure 2D) were reduced in amitriptyline-treated animals compared to control animals. Using a two-sided statistical test, these differences did not reach statistical significance. However, based on our *a priori* hypothesis, a one-sided *t*-test revealed significant reductions in both eosinophils and T cells.

To assess T cell activation, the typical type 2 cytokines IL-4 and IL-5 were measured in the BALF of amitriptyline-treated animals and controls. While no significant difference in IL-4 (Figure 2E) levels was observed, the amitriptyline-treated animals showed a trend towards lower IL-5 (Figure 2F) levels. This indicates that amitriptyline modulates the type 2-driven inflammatory response in the OVA model, most prominently reflected by a reduction in eosinophils and T cells.

### 3.3. Inhaled Amitriptyline Preserves Bronchodilatory Efficacy but Exhibits Blunted Immunomodulatory Activity in a Therapeutic HDM-Induced Model of AAI

Based on the improvement in lung function and the reduction in inflammatory cells in the OVA model, we next investigated whether similar effects occur in the clinically more relevant therapeutic HDM model. Specifically, we tested a therapeutic paradigm where inhalation of amitriptyline only during the phase of HDM provocation (therapeutic model, starting at day 7) but not during sensitization leads to relevant changes.

**Figure 3:**
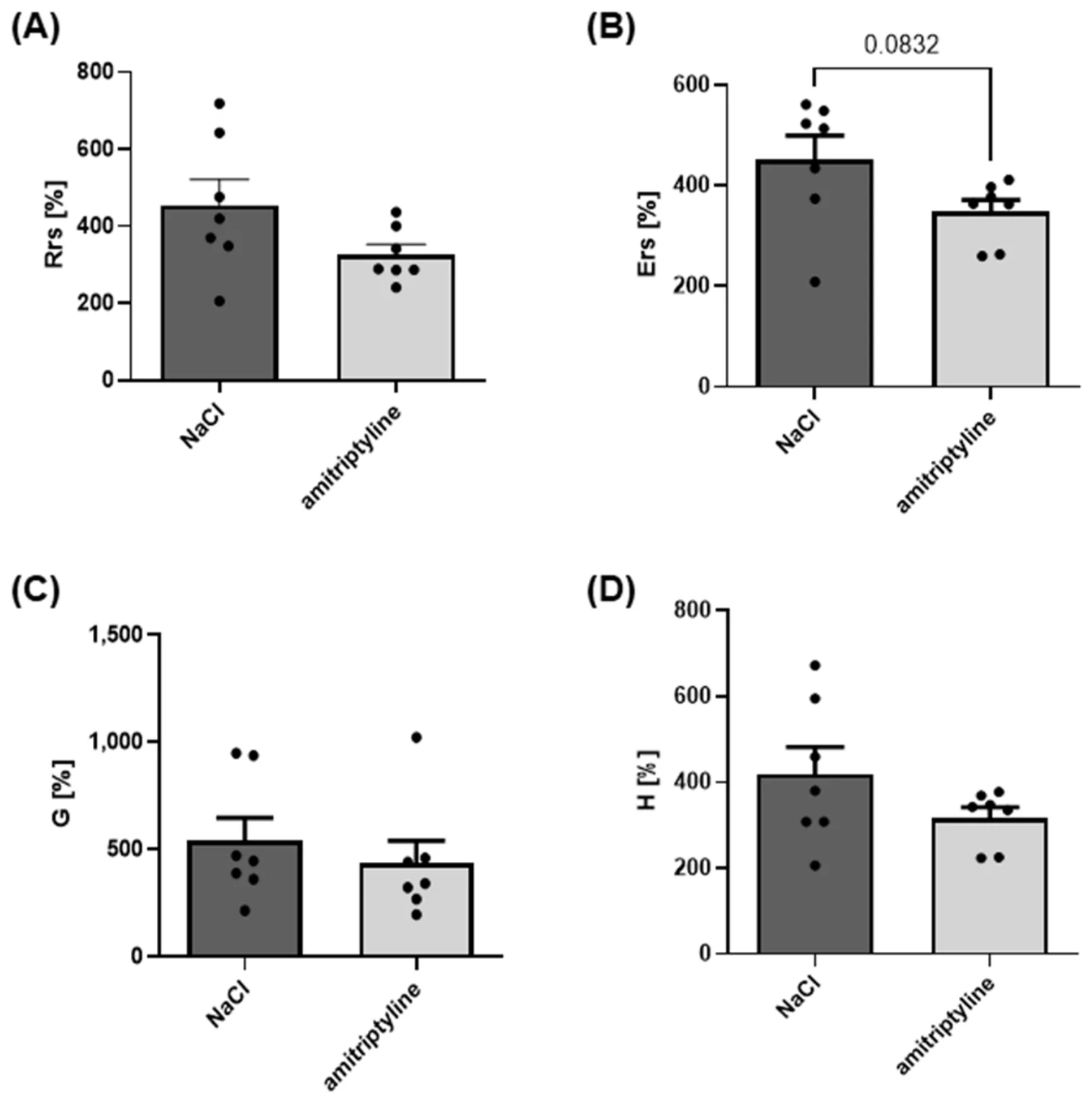
Lung function parameters of house dust mite (HDM)-sensitized animals were measured with the FlexiVent^®^ system after application of 1 mg/mL acetylcholine (Ach). All values were normalized on basal ventilation values. (**A**) Total lung resistance Rrs (*p* = 0.1128) {*p* = 0.05}, (**B**) elastance Ers (*p* = 0.0832) {*p* = 0.0416}, (**C**) tissue resistance G (*p* = 0.5095) {*p* = 0.2548}, and (**D**) tissue elastance H (*p* = 0.1748) {*p* = 0.08} of HDM-sensitized mice, nebulized with 0.9% NaCl or 3.3 mg/mL amitriptyline, were measured. The data were tested for normal distribution and analyzed using a two-sided *t*-test. All graphs represent means ± SEM. *n* = 7 in both groups. Each dot represents one mouse.

Again, total resistance (Rrs), total elastance (Ers), tissue resistance (G), and tissue elastance (H) of the airways were decreased in amitriptyline-treated animals (Figure 3). Using a two-sided statistical test, these differences did not reach statistical significance. However, based on our *a priori* hypothesis of a protective effect of amitriptyline, a one-sided *t*-test revealed significant reductions in Rrs and Ers. For AHR induction in the HDM model, only acetylcholine concentrations up to 1 mg/mL were used, as animals already showed a strong response to this concentration, which was comparable to the response observed at 3.16 mg/mL in the OVA-induced murine model of AAI.

**Figure 4:**
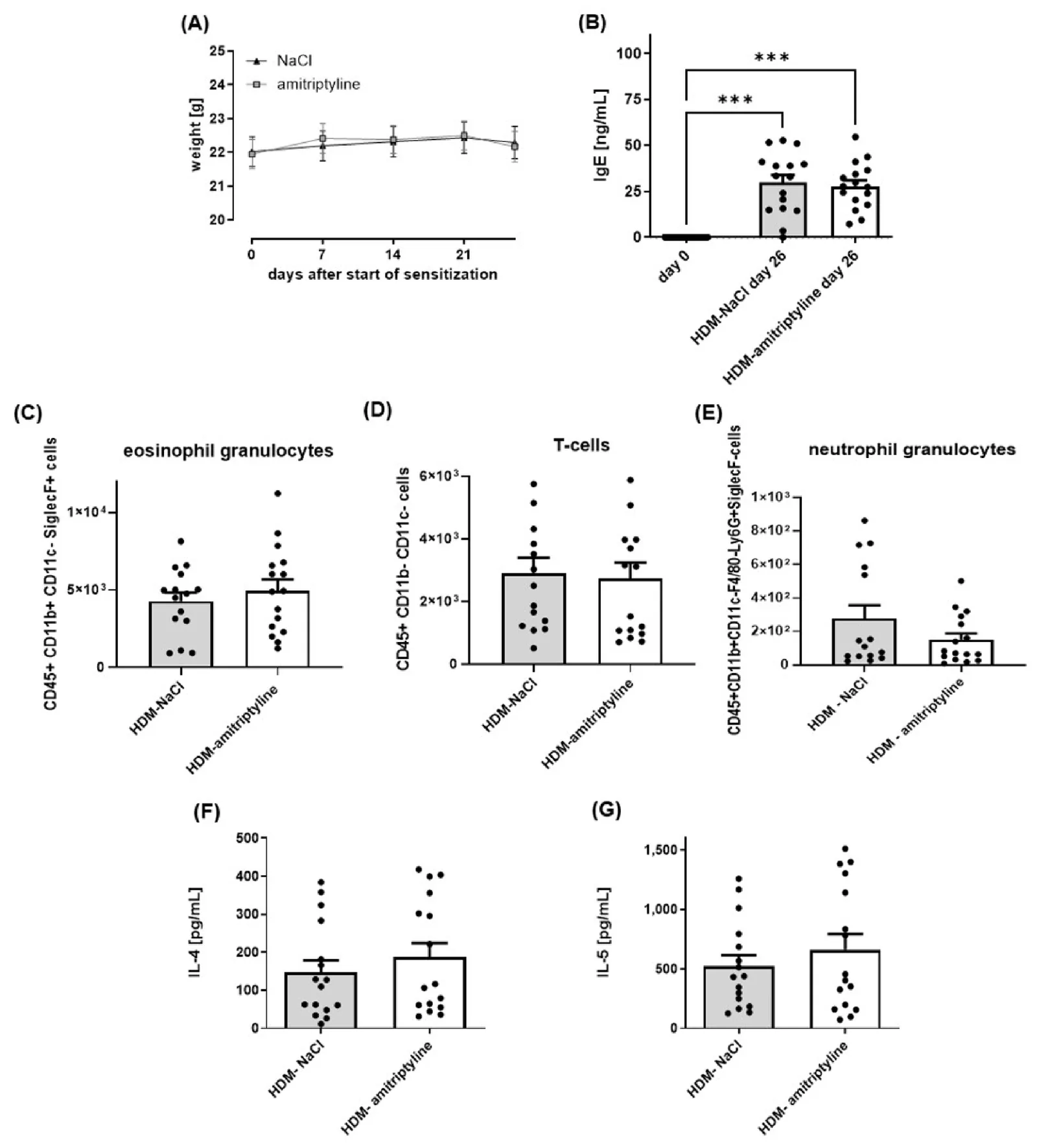
Immunomodulatory effects of amitriptyline in the house dust mite (HDM)-induced murine model of AAI. (**A**) Effect of inhaled amitriptyline on the weight of animals during the time of sensitization (*p* = 0.9220). (**B**) Significant increase in IgE serum levels of HDM-sensitized mice (*p* = <0.001). Counts of (**C**) eosinophilic granulocytes (*p* = 0.4452) {*p* = 0.1076}, (**D**) T cells (*p* = 0.8184) {*p* = 0.3519}, and (**E**) neutrophil granulocytes (*p* = 0.1656) {*p* = 0.08} in the bronchoalveolar lavage fluid (BALF) of HDM-sensitized animals ± amitriptyline. (**F**) IL-4 (*p* = 0.4323) {*p* = 0.2161} and (**G**) IL-5 cytokine levels (*p* = 0.3995) {*p* = 0.1997} of the BALF. The data were tested for normality: (**A**) was analyzed using a two-way ANOVA, (**B**) was analyzed using a one-way ANOVA, and (**C**–**G**) were analyzed using a two-sided *t*-test. All graphs represent means ± SEM; *** *p* ≤ 0.001. *n* = 16 in all groups. Each dot represents one mouse.

Again, body weight again did not differ between the groups over the 26-day treatment period (Figure 4A).

Successful induction by AAI was confirmed by elevated serum IgE levels. IgE serum levels were significantly increased in HDM-sensitized animals compared to day 0, with no difference between amitriptyline-treated animals and controls (Figure 4B). Concurrently, while the absolute number of eosinophilic granulocytes (Figure 4C) and T cells (Figure 4D) in BALF increased in the murine model of AAI, inhalation of amitriptyline did not influence this phenotype. Analysis of neutrophilic granulocytes revealed a trend towards decreased levels in animals treated with amitriptyline (Figure 4E). Type 2-associated cytokines IL-4 and IL-5 in lung tissue and BALF revealed a tendency towards reduced levels in animals nebulized with amitriptyline (Figure 4F,G)). In summary, amitriptyline again improved lung functions in the HDM model, whereas immunomodulatory effects were less pronounced than in the OVA model.

### 3.4. Amitriptyline Treatment Does Not Directly Alter Human T_H_2 Cell Activation or Cytokine Profiles In Vitro

**Figure 5:**
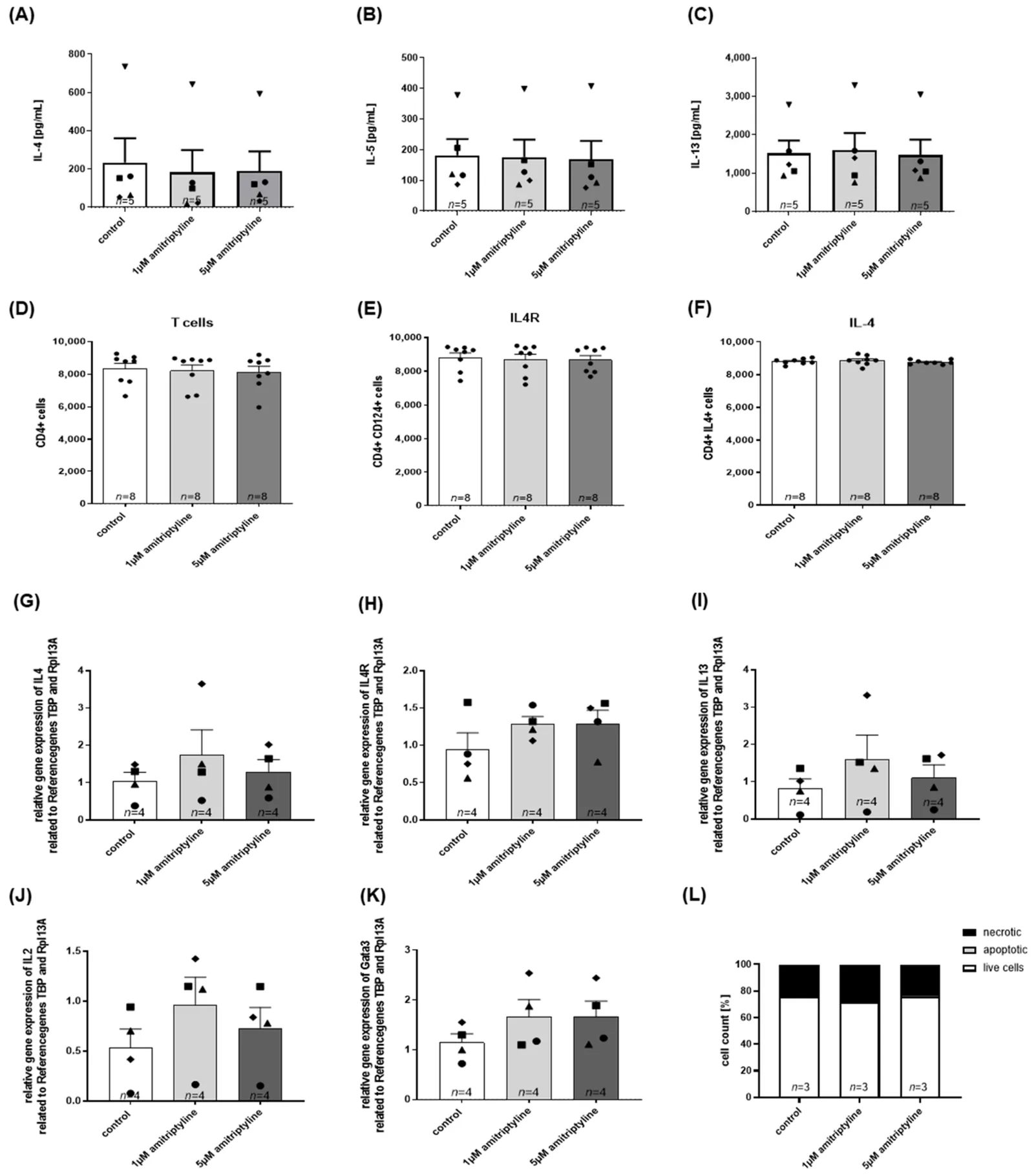
Release of cytokines and expression of mRNA in human differentiated Th2 cells treated with different amitriptyline concentrations (1 µM, 5 µM). Human T cells were differentiated into Th2 cells and treated with different amitriptyline concentrations for 24 h. (**A**) IL-4 (*p* = 0.9446), (**B**) IL-5 (*p* = 0.9853), and (**C**) IL-13 (*p* = 0.9751) cytokine levels of the cell supernatant. Flow cytometry data of (**D**) T cells (*p* = 0.9065), (**E**) the IL4 receptor (*p* = 0.9202), and (**F**) IL4 (*p* = 0.6162). Gene expression levels of (**G**) *Il4* (*p* = 0.6255), (**H**) *Il4R* (*p* = 0.3226), (**I**) *Il13* (*p* = 0.7837), (**J**) *Il2* (*p* = 0.8994) and (**K**) *Gata3* (*p* = 0.3592). (**L**) Live cells as a percentage after stimulation with different concentrations of amitriptyline. The data were tested for normality and analyzed using a one-way ANOVA. All graphs represent means ± SEM.

In previous studies, we demonstrated that amitriptyline affects bronchoconstriction in an ex vivo model of human precision-cut lung slices [19]. To assess the translational relevance of the immunomodulatory effects observed in the OVA model, we next investigated the effect of amitriptyline on human T_H_2 lymphocytes. Cytokine analysis of the cell culture supernatants IL-4 (Figure 5A), IL-5 (Figure 5B), and IL-13 (Figure 5C), flow cytometry of T cells (Figure 5D), the IL-4 receptor (Figure 5E), and intracellular IL-4 (Figure 5F), and type 2-associated gene expression of *Il4* (Figure 5G), the *Il4 receptor* (Figure 5H), *Il13* (Figure 5I), *Il2* (Figure 5J), and *Gata3* (Figure 5K) showed no significant differences between amitriptyline-treated and control cells, despite variability between individual donors.

Amitriptyline has been shown to induce oxidative stress in a dose-dependent manner, which can result in apoptosis and reduced cell proliferation [31]. In our setup, cell viability was maintained across all tested concentrations, with approximately 80% of cells remaining viable compared to untreated controls (Figure 5L).

In summary, amitriptyline did not have an immunomodulatory effect in human T_H_2 lymphocytes under these experimental conditions.

**Figure 6:**
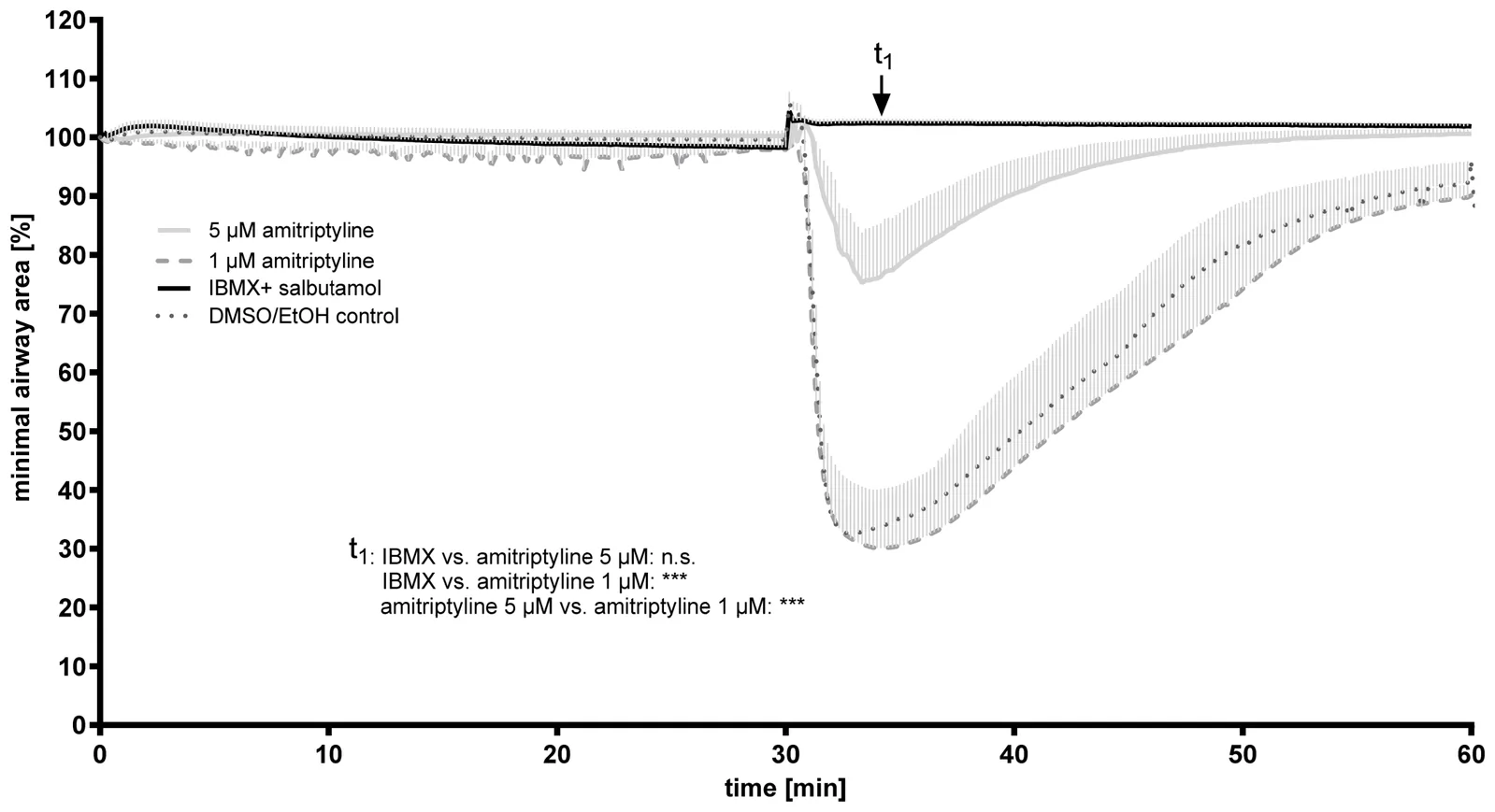
Passive sensitization of rat PCLSs treated with different concentrations of amitriptyline. Statistical analysis was performed at time point t_1_ (*p* = <0.001). Data were checked for normal distribution and analyzed using a one-way ANOVA. All graphs represent means ± SEM; *** *p* ≤ 0.001. *n* = 3 in all groups. n.s. indicates non-significant differences.

Based on previous findings by Hempel et al. [19], showing that amitriptyline strongly inhibits bronchoconstriction and promotes bronchodilation in PCLSs, we additionally investigated the effect of amitriptyline in a model of passive OVA sensitization. In passive sensitization, lung slices from unsensitized animals are incubated ex vivo with serum from sensitized animals, which contains allergen-specific IgE antibodies that bind to mast cell FcεRI receptors, priming them for allergen-induced activation. In our setup, OVA stimulation of sensitized control slices induced a significant contraction (Figure 6). In contrast, the addition of IBMX/salbutamol, used as a bronchodilator (positive control), completely prevented the airway contraction. Pre-incubation with low-dose amitriptyline (1 µM) did not affect airway contraction, whereas 5 µM amitriptyline significantly attenuated OVA-induced bronchoconstriction. These data demonstrate that amitriptyline effectively counteracts allergen-triggered, mast-cell-mediated smooth muscle contraction in passive OVA sensitization.

## 4. Discussion

In this study, we investigated the effects of inhaled amitriptyline across distinct experimental models of allergic asthma. In addition, we assessed its effects on human T_H_2 lymphocytes and passively sensitized precision-cut lung slices (PCLSs). Overall, our data demonstrate that localized amitriptyline administration consistently improves dynamic lung mechanics in vivo and ex vivo, whereas its anti-inflammatory efficacy is highly dependent upon the specific murine model of AAI and the experimental timeline.

### 4.1. Context-Dependent Immunomodulatory Effect of Inhaled Amitriptyline in Murine Models of AAI

In our OVAmurine model of AAI, amitriptyline improved lung functions and reduced the number of eosinophils and T cells in BALF. In this model, amitriptyline was administered prophylactically during the early phases of immune priming before sensitization (starting at day 1), whereas the HDM model reflects a therapeutic intervention in an already established inflammatory milieu (starting at day 7). Amitriptyline improved lung functions in both models but reduced inflammatory cell infiltration only in the OVA model, while type 2-associated cytokine levels in the HDM model remained largely unchanged. The ovalbumin model is a well-established experimental model of allergic asthma, typically performed using an adjuvant such as aluminum hydroxide [24]. Ovalbumin, a prominent protein allergen derived from chicken egg whites, induces reproducible, robust eosinophil airway inflammation and type 2 immune polarization characteristic in pediatric atopic asthma phenotypes [32,33]. House dust mite allergens are clinically relevant aeroallergens that can induce allergic airway inflammation without exogenous adjuvants in all age groups, thereby more accurately recapitulating human disease pathophysiology [24]. In contrast, HDM exposure induces a more complex immune response with broader but often less pronounced immune cell activation [23,24,25,34]. Consistent with this, amitriptyline improved lung function parameters in the HDM model, similarly to the OVA model, yet it failed to significantly alter global inflammatory cell influx or type 2 cytokine production. Interestingly, a trend towards lower neutrophil numbers was observed for the amitriptyline-treated animals, which may reflect the more heterogeneous inflammatory profile typically associated with HDM-driven airway disease [35]. Therefore, these findings suggest that the immunomodulatory effects of amitriptyline may be restricted to early inflammatory phases or clearly type 2-driven models.

**Figure A5:**
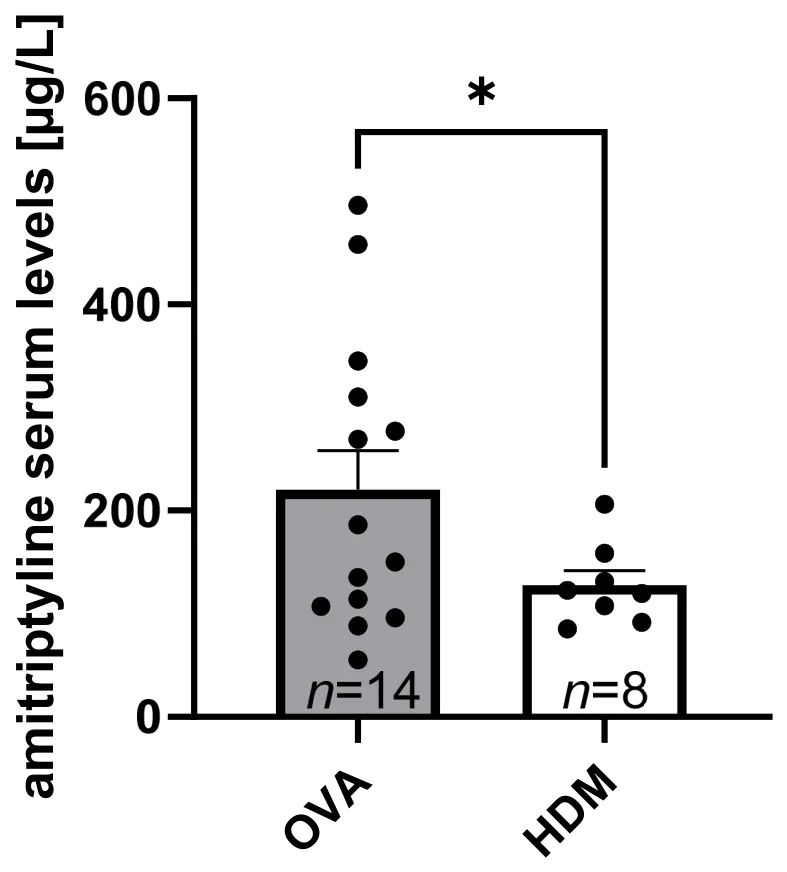
Amitriptyline serum levels of mice treated with the OVA-induced model of allergic airway inflammation (AAI) versus the HDM-induced model of AAI. The data were tested for normal distribution and analyzed using a two-sided *t*-test. All graphs represent means ± SEM, * *p* ≤ 0.05. Each dot represents one mouse.

Beyond the fundamental differences in model design, the different immunological outcomes may also be linked to the systemic exposure levels of amitriptyline. Serum levels of amitriptyline were notably lower in the therapeutic HDM model (127.9 µg/L ± 13.82 µg/L) compared to the prophylactic OVA model (220.4 µg/L ± 37.76 µg/L) (see Appendix C, Figure A5). This may reflect the shorter treatment duration (26 vs. 35 days) and the later onset of administration. In addition, inflammation-dependent changes in epithelial integrity may have influenced local drug uptake and distribution, as epithelial damage may alter the penetration of lipophilic drugs [36,37]. It remains to be investigated whether a prophylactic treatment design in the HDM model would lead to similar immunological outcomes. Together, these findings indicate that while immunological effects of amitriptyline vary depending on timing and model complexity, the improvement in lung function is a robust and consistent effect across experimental conditions.

Moreover, the early-phase response plays a central role in bronchial asthma, particularly in acute allergen-induced bronchoconstriction. Our passive sensitization PCLS model allows the investigation of direct pharmacological effects on airway contractility independent of systemic immunity [38]. Amitriptyline dose-dependently inhibited allergen-induced airway contraction, supporting a direct pharmacological effect on airway smooth muscle contractility downstream of allergen-induced mediator release.

### 4.2. Impact of Amitriptyline on Primary Human T_H_2 Lymphocytes

To investigate the direct effects of amitriptyline on human T_H_2 lymphocytes, we examined differentiated T_H_2 cells under amitriptyline treatment. Previous studies have demonstrated that amitriptyline modulates T cell function in humans, although in a non-asthmatic context. For instance, it has been shown that amitriptyline modulates the phenotype of human peripheral blood T cells, resulting in a decreased number of IFN-γ and IL-17-producing T cells [39]. Similarly, a clinical study in patients with chronic pain reported that systemic amitriptyline treatment was associated with an increased frequency of regulatory T cells and a reduction in effector T cells. This shift of T cell populations was accompanied by decreased levels of pro-inflammatory cytokines such as IL-6 and TNF-α, while anti-inflammatory cytokines, including IL-10, were elevated [40]. One possible mechanistic pathway involves the inhibition of acid sphingomyelinase (ASM), as amitriptyline is a well-known functional inhibitor of this enzyme. In this context, Böll et al. demonstrated that ASM inhibition by amitriptyline can reduce IL-4 release from murine T lymphocytes in vitro [16]. Since IL-4 is also produced in innate immune cells such as basophils and ILC2s, the lack of a detectable effect in differentiated T_H_2 lymphocytes does not exclude immunomodulatory actions of amitriptyline on other immune cell populations [6,7,8]. These cell types were not investigated in the present study and should therefore be addressed in future studies. Overall, our findings suggest that amitriptyline has no major direct effect on differentiated human T_H_2 lymphocytes under the experimental conditions tested. Importantly, our study represents the first dedicated investigation of primary T_H_2 cells in this context. Future studies should further investigate whether amitriptyline influences epigenetic regulation of type 2 immune cells or other inflammatory cell populations, which may contribute to the context-dependent immunomodulatory effects in the present study [41,42].

### 4.3. Direct Mechanism of Amitriptyline-Mediated Bronchoconstriction

The consistent improvement in lung functions across different murine models of AAI suggests a direct anti-obstructive effect of amitriptyline on airway contractility. In previous studies, we demonstrated that this anti-obstructive effect is ASM-independent, as amitriptyline still improved lung function in ASM-knockout mice as well as in controls [19]. These findings are in line with ex vivo experiments of Hempel et al. and Klein et al., demonstrating that amitriptyline directly inhibits bronchoconstriction and promotes airway dilatation in PCLSs [19,43]. In contrast to classical receptor binding screens conducted in the 1980s and earlier, modern tracking has failed to identify major competitive antagonism activity of amitriptyline on classical signaling pathways involved in bronchoconstriction [44,45]. Recent findings suggest that these effects may involve the disruption of lipid rafts and caveolae in airway smooth muscle cells and epithelial cells, potentially altering signaling cascades involved in contraction [43]. However, the precise molecular mechanism remains to be fully elucidated.

### 4.4. Limitations of the Study

First, the findings are based on a murine model of ovalbumin-induced allergic asthma, which reproduces important features of allergic airway inflammation but does not fully reflect the heterogeneity and complexity of human asthma. Therefore, the translational relevance of these findings requires confirmation in additional preclinical models and ultimately in clinical studies.

Second, only one dosing regimen and treatment duration were investigated. Future studies should evaluate dose–response relationships, different treatment schedules, and the long-term effects of inhaled amitriptyline.

Third, although inhaled amitriptyline improved airway function and modulated inflammatory parameters, the precise cellular and molecular mechanisms underlying these effects remain to be elucidated.

Finally, while the inhaled route aims to maximize local pulmonary effects and minimize systemic exposure, a comprehensive assessment of systemic pharmacokinetics and potential off-target effects was beyond the scope of the present study.

### 4.5. Potential Clinical Application and Future Directions

Amitriptyline is a tricyclic antidepressant that has been in clinical use since the early 1960s, primarily for the treatment of major depressive disorder and chronic pain syndromes, including neuropathic pain, migraine prophylaxis, and fibromyalgia [10,30,45]. Beyond this, amitriptyline acts as a functional inhibitor of ASM, thereby modulating ceramide metabolism. The first clinical evidence for a beneficial pulmonary effect of amitriptyline emerged from studies in cystic fibrosis (CF). Preclinical data have demonstrated that amitriptyline, through ASM inhibition, significantly reduces neutrophilic infiltration and reduces *Pseudomonas aeruginosa* lung infection in CF mice [21]. Translating these findings into a clinical setting, Riethmüller et al. conducted a randomized pilot study in four adult CF patients receiving amitriptyline twice daily for 14 days. Amitriptyline treatment here showed a relative FEV_1_ improvement of 14.7 ± 5% [46]. In line with this, in 2013, Nährlich et al. reported that long-term therapy of cystic fibrosis patients with oral and therefore systemic low-dose amitriptyline improves FEV_1_ by 7.6 ± 7.0%, whereas FEV_1_ decreased in the control group. The investigators concluded that this clinical stabilization was directly mediated by the effect of amitriptyline on ceramide levels [47]. Notably, this improvement in lung function is comparable to improvements reported for novel CFTR modulators such as elexacaftor/tezacaftor/ivacaftor, which improved FEV_1_ by approximately 11–13%, underscoring the broader therapeutic potential of amitriptyline in pulmonary disease [48]. Beyond CF, early clinical observations in case reports also suggested a role for amitriptyline in allergic airway disease [11].

Taken together, our findings identify inhaled amitriptyline as a promising therapeutic candidate, combining direct anti-obstructive effects on airway smooth muscle contractility with immunomodulatory properties that depend on the inflammatory context and treatment timing. Future studies should further characterize the effects of amitriptyline on other T cell subtypes and other immune cell populations to further investigate the immunomodulatory mechanism of amitriptyline.

## 5. Conclusions

This study identifies inhaled amitriptyline as a viable therapeutic strategy for bronchial asthma, effectively mitigating airway hyperresponsiveness across varying inflammatory backgrounds. Our findings demonstrate that amitriptyline consistently improves lung mechanics in both prophylactic (OVA) and therapeutic (HDM) murine models. Although its immunomodulatory effects were more pronounced when initiated during the early sensitization phase, the bronchodilatory effect remained robust across all conditions. Furthermore, the absence of systemic side effects underscores the safety of targeted pulmonary delivery. Taken together, these results support the repositioning of amitriptyline for asthma management. Future studies using subchronic and chronic allergen exposure models—characterized by structural airway remodeling and complex immune cascades—are warranted to fully elucidate the long-term therapeutic potential of inhaled amitriptyline.

## Data Availability

All data generated or analyzed during this study are included in this published article.

## Abbreviations

The following abbreviations are used in this manuscript:

AAI	Allergic airway inflammation
ACh	Acetylcholine
AHR	Airway hyperresponsiveness
Alu	Aluminum hydroxide
ASM	Acid sphingomyelinase
BALF	Bronchoalveolar lavage fluid
CF	Cystic fibrosis
Ers	Total respiratory system elastance
FEV1	Forced expiratory volume in 1 s
G	Tissue resistance
H	Tissue elastance
HDM	House dust mite
IgE	Immunoglobulin E
IL	Interleukin
i.p.	Intraperitoneal
OVA	Ovalbumin
PBS	Phosphate-buffered saline
PCLS	Precision-cut lung slice
Rrs	Total respiratory system resistance
T_H_2	T helper type 2 (cells)

## Appendix B

**Table A1:** qPCR Primer sequences.

Primer	Sequences
*Il4*	Fwd: CTGTGCTCCGGCAGTTCTAC
	Rev: TCACAGGACAGGAATTCAAGC
*Il4 receptor*	Fwd: AAACGACCCGGCAGATTTCA
	Rev: AGACGGCCAGGATGACAATG:
*Il13*	Fwd: AGGTCTCAGCTGGGCAGTTTTC
	Rev: AGCAGGTCCTTTACAAACTGGG
*Il2*	Fwd: CAAGAATCCCAAACTCACCAGG
	Rev: ATGTTTCAGTTCTGTGGCCTTC
*Gata3*	Fwd: AACATCGACGGTCAAGGCAA
	Rev: CACACCTGGCTCCCGTG
